# Supporting senior high-school students’ measurement and geometry performance: Does spatial training transfer to mathematics achievement?

**DOI:** 10.1007/s13394-022-00416-y

**Published:** 2022-04-25

**Authors:** Jonathan Adams, Ilyse Resnick, Tom Lowrie

**Affiliations:** University of Canberra, Canberra, Australia

**Keywords:** Spatial reasoning, Mathematics achievement, Measurement and geometry, Classroom-based intervention, Pedagogical framework

## Abstract

It is well established that spatial reasoning skills (i) support mathematics achievement, (ii) are malleable, and (iii) can be improved through training. More recently, there has been interest in using spatial training to causally support corresponding improvements in mathematics achievement; however, findings so far appear to be mixed. The current study explores the effect of a spatial reasoning intervention on Year 11 students’ spatial reasoning skills and mathematics achievement and considers the role of a pedagogical framework and the multidimensional nature of mathematics and spatial reasoning in the design of the intervention. The Experience-Language-Pictorial-Symbolic-Application (ELPSA) pedagogical framework was used to modify an existing spatial intervention program for delivery by high-school educators to Year 11 students (an important but understudied population). The spatial intervention involved training a range of spatial skills over an extended timeframe. Students were randomly assigned to the intervention condition or to a business-as-usual control (*n* = 73). Using a pre-/post-test design, we found the intervention was successful in improving participants’ spatial reasoning skills and performance on measurement and geometry items compared to the control condition but not on number and algebra items. These findings demonstrate that spatial training can support mathematics achievement in certain contexts, highlighting the importance of identifying how individual spatial skills support specific mathematics tasks. Consideration was given for how to use strong pedagogical techniques to scaffold transfer, finding utility in the ELPSA framework. Implications for how to embed spatial training within real mathematics classrooms, as done in the current study, are discussed.

## Introduction

It is widely understood that spatial reasoning supports mathematical achievement (e.g. Landy & Goldstone, 2010; Mix et al., 2016; Thompson et al., 2013). This connection is present across different ages and across different mathematical and spatial tasks (e.g. Burnett et al., 1979; Delgado & Prieto, 2004; Lubinski & Benbow, 1992; Nuttall et al., 2005). Spatial skills are highly predictive of the pursuit of STEM careers and overall success in STEM subjects, particularly mathematics (Wai et al., 2009). More recently, there is a growing body of evidence demonstrating that teaching spatial skills causally improves mathematics achievement (e.g., Cheng & Mix, 2014; Lowrie et al., 2017, 2018a; Sorby et al., 2013; Sorby & Veurink, 2019). Studies are varied, however, with some showing broad transfer (e.g. Lowrie et al., 2017, 2019), some finding narrow transfer (Cheng & Mix, 2014; Gilligan et al., 2020), and still, others finding no evidence of improved mathematics achievement in response to spatial training (e.g. Hawes et al., 2015). In the following sections, we account for such mixed findings by considering (1) the multidimensional nature of both spatial reasoning and mathematics and (2) the role and nature of pedagogical frameworks, particularly the experience-language-pictorial-symbolic-application (ELPSA) framework (Lowrie et al., 2018b) in supporting spatial learning. Building from this research, the current study examines the efficacy of a spatial reasoning intervention on Year 11 students’ spatial reasoning skills and mathematics achievement.

## Literature review

### Defining spatial reasoning and mathematics

Spatial reasoning and mathematics are both complex and multidimensional involving a wide range of interconnected skills. Mathematics is a broad area of study, encapsulating a range of different strands, such as number, algebra, measurement, and geometry (Australian Curriculum, Assessment and Reporting Authority (ACARA), 2010). Although many of these strands are interconnected, the types of skills required to solve any given problem will vary depending on the topic. For example, interpreting a diagram representing geometric information requires different underlying skills than those used in solving an algebraic equation (Geary et al., 2015). The same problem may also require different or additional skills depending on the way the problem is framed. For example, Duffy et al. (2020) found that the ability to apply the necessary algebraic skills was not sufficient for participants to solve mathematical tasks presented as word problems. As well as procedural fluency, word problems often require additional literacy and interpretation skills to arrive at a correct solution.

Similarly, spatial reasoning is comprised of many different, yet interrelated, individual spatial skills (Buckley et al., 2018; Newcombe & Shipley, 2015). For example, being able to imagine an object rotating is a separable skill from being able to imagine someone else’s perspective (Hegarty & Waller, 2004). The relationship between individual spatial skills is complex, and consequently, the field has not reached an agreed upon framework for identifying and characterising spatial reasoning skills (e.g. Carroll, 1993; Linn & Petersen, 1985; Miyake et al., 2001; Newcombe & Shipley, 2015). Indeed, Buckley et al. (2018) suggest there are likely many more unique spatial reasoning skills not currently included in existing frameworks.

One spatial reasoning framework particularly relevant for considering how to connect spatial skills with classroom practice is Ramful et al.'s (2017) because they identify three spatial reasoning constructs that are closely aligned with activities described in mathematics curricula, namely mental rotation, spatial orientation, and spatial visualisation. Mental rotation relates to imagining the rotation of an object in two- or three-dimensional space. Spatial orientation involves imagining how a scene might appear from a different perspective. Mental rotation and spatial orientation are well defined within the literature and have been shown to be separable spatial skills (Hegarty & Waller, 2004; Kozhevnikov & Hegarty, 2001). Spatial visualisation, under Ramful et al.'s (2017) framework, is a broader construct, encompassing challenging tasks that require reasoning about changing spatial relationships within and between objects over time. This framework has been used in several studies exploring the causal relationship between spatial reasoning interventions and improvements in mathematics (e.g. Lowrie et al., 2017, 2019).

### The effect of spatial training on mathematics achievement

Many studies have shown that spatial reasoning skills are malleable and can be improved through training in a wide range of populations and types of training (see Uttal et al., 2013 for a meta-analytic review). Importantly, the training of spatial skills can lead to corresponding improvements in mathematics achievement (e.g. Lowrie et al., 2017, 2018a; Sorby et al., 2013; Sorby & Veurink, 2019); however the extent to which this occurs varies according to the age of participants, the design of the intervention program, and the extent to which the mathematical measures are aligned to the skills trained (Hawes et al., 2022).

Approaches demonstrating limited or narrow transfer of spatial training to mathematics achievement tend to be narrow in scope and short in duration (e.g. Cheng & Mix, 2014; Hawes et al., 2015). By contrast, the most promising approaches, demonstrating broad transfer of spatial training to general mathematics achievement, have involved training a wide range of spatial skills over an extended timeframe alongside regular mathematics learning (e.g. Hawes et al., 2017; Lowrie et al., 2019). For example, Sorby et al. (2013) delivered a 15-week spatial reasoning intervention that included mental rotation, perspective taking (referred to in the current study as spatial orientation), and a range of complex spatial visualisation skills. The study found the spatial reasoning intervention led to improvements in students’ calculus grades. Continuation of the program in future years also led to improved grades across a range of undergraduate subjects and higher retention rates throughout the engineering program (Sorby et al., 2018).

This is certainly promising; however, Sorby et al.'s (2013, 2018) work is focused on first-year engineering students, who represent a biased population. Since these students have gained entry into an undergraduate engineering program, they are likely to have significantly higher mathematics skills than the general population. This high level of mathematical competence may have supported the transfer of spatial training to mathematics in a way that may not be representative of the general population. For example, although students’ interpretation and understanding of some types of mathematical problems can benefit from spatial training, it does not lead to overall improved performance when students lack the requisite procedural mathematics skills (Duffy et al., 2020).

As a result, to engage with programs such as Sorby et al. (2013, 2018), students without a strong mathematical or spatial background may require additional scaffolding and support to meaningfully engage with the program. Here, we detail ELPSA pedagogical framework (Lowrie et al. 2018b) as a way to provide this scaffolding. As spatial reasoning is the foundation for two of the stages (language and symbolic), it provides the opportunity for explicit links to be made between spatial reasoning and mathematics. In addition, educators administering a spatial intervention may also benefit from having pedagogical framing (Lowrie et al., 2018a) because secondary STEM educators are likely to have relatively higher spatial skills (Atit et al., 2018) and subsequently may not have intuitions around the hurdles low-spatial reasoning students may encounter.

### The ELPSA pedagogical framework

The ELPSA framework has been designed to support planning, teaching, and evaluation of learning in mathematics classrooms (Lowrie et al., 2018b). Based on constructivist learning theory (Liebeck, 1984; Vygotsky, 1978; Wenger, 1999), ELPSA promotes student learning by guiding students through five distinct stages (see Table 1 for an overview) that introduce new ideas through tangible, concrete experiences, and scaffolding students towards applying their understanding in more novel, abstract contexts. The ELPSA framework is particularly effective for supporting learning across all ages and skill levels because it implicitly requires the educator to consider the differential pedagogical needs of the class when delivering learning materials. Since heavy importance is placed on the past experiences of students when considering how to introduce new ideas, the kinds of activities incorporated into each stage will vary depending on the age and skill level of the students.

**Table 1:** Stages of the ELPSA framework adapted from Lowrie et al., (2018b)

Stages	Principles
Experience	Evoke out-of-school experience to build on understanding Reinforcing existing understandings to new concepts For new concepts, provide physical experiences where possible
Language	Reinforce mathematics terminology throughout the lesson Foster conversations that link experiences with language. Build bridges between experience and language Encourage students’ own language while modelling precise terminology
Pictorial	Includes concrete manipulatives, external representations, and students’ encoded understandings Ensure multiple representations are provided including non-prototypical representations Progressively model effective pictorial heuristics
Symbolic	Introduce symbolic expressions alongside pictorial representations Encourage multiple appropriate symbolic representations Model fluency and flexibility with efficient symbolic representations
Application	Apply symbolic reasoning to real-life situations Apply symbolic reasoning to related mathematics concepts Consider the application of the mathematics concepts outside the classroom

The ELPSA framework has been used previously to assist students’ spatial learning in both elementary and high-school contexts (Lowrie et al., 2018a, 2019). Many existing spatial training studies have researchers administer the intervention directly to students. By contrast, these studies had teachers incorporate spatial training within their regular classroom practice. In both cases, teachers were provided with professional learning around the ELPSA framework, and lessons were designed to move students through each stage. This approach maintained experimental fidelity while still allowing teachers to adapt the lessons to meet the pedagogical needs of their particular students. In both studies, the interventions led to improvements in students’ spatial reasoning skills.

The ELPSA framework is also an effective tool for analysing existing programs and identifying their appropriateness for a given population. Take, for example, Sorby et al.'s (2013, 2018) highly successful spatial reasoning intervention program, which was tailored for undergraduate engineering students who likely already have an extensive background in mathematics. As is appropriate for such a group, concepts and definitions are introduced only formally (e.g. presented beside diagrams); students do not engage in lessons that explicitly target language learning, moving on to the pictorial and symbolic stages, with few opportunities to articulate their understandings in their own words. This is justified, given these students likely have some prior knowledge of mathematical concepts as well as experience in mathematics classes where terms have been introduced in a similar fashion.

Although such an approach is appropriate for undergraduate engineering students, the ELPSA framework suggests that younger populations and individuals with less experience with mathematics may require more opportunities to develop experiences with these basic skills and concepts as well as language to support more complex learning. Specifically, modifications using the ELPSA framework might focus on introducing concepts with more concrete experiences, which explicitly require students to develop and use spatial language.

Sorby et al.'s (2013, 2018) program provides strong scaffolding for representing spatial concepts pictorially, using diagrams to illustrate key ideas and providing step-by-step guidance through worked examples. Symbolic representations are introduced and supported pictorially, and work-sample exercises give students the opportunities to apply their spatial understanding to a range of problems. Nevertheless, younger and less mathematically experienced populations may benefit from more explicit scaffolding to support students in understanding pictorial and symbolic representations. A summary of this analysis is provided in Table 2.

**Table 2:** Analysis of Sorby et al. (2013, 2018) program using the ELPSA framework

ELPSA element	Sorby et al. (2013, 2018) program
Experience	Built on students’ past learning of mathematics Assumption of familiarity with mathematical and spatial language
Language	Definitions of key terms provided next to diagrams Limited opportunities for students to articulate understanding
Pictorial	Key ideas illustrated through diagrams Step-by-step guidance through worked examples Relies on the ability to interpret 2-D representations of 3-D objects
Symbolic	Introduced clearly alongside pictorial representations
Application	Follow naturally from prior learning Explore a range of contexts

### Present study

The present study aims to explore the effect of a spatial reasoning intervention on Year 11 students’ spatial reasoning skills and mathematics achievement. This is an important population to study since for many students the last 2 years of high school are the final opportunity to engage in formal mathematical study. Consequently, the experiences they have at this stage of schooling will shape their beliefs and attitudes towards mathematics for the rest of their lives. Due to the success of Sorby et al.'s (2013, 2018) program in improving the spatial reasoning and mathematical achievement of students who were only slightly older, the present study modifies the program for use with this new population.

Recall that both mathematics and spatial reasoning involve multidimensional constructs. We considered the multi-dimensionality of spatial reasoning by training several spatial skills as well as using a composite spatial reasoning score that includes three spatial reasoning constructs identified by Lowrie and colleagues (Lowrie et al., 2017; Ramful et al., 2017). We considered the multidimensionality of mathematics by examining the effects of the intervention on two mathematics strands identified by the Australian Curriculum: Mathematics (ACARA, 2010)—Number and Algebra, and Measurement and Geometry. The ELPSA framework (Lowrie et al., 2018b) is used to guide these modifications to make the existing program accessible to this younger group of participants who likely do not have the same background and skills in mathematics.

Distinct from existing research demonstrating transfer between spatial training and mathematics understanding (e.g. Cheng & Mix, 2014; Gilligan et al., 2020), the current study maintains a high level of ecological validity by developing intervention materials to be delivered by classroom teachers during regular mathematics lessons.

## Methods

### Participants and setting

Participants for the study were Year 11 students from two senior secondary schools in a large Australian city with middle-to-high socioeconomic status (SES; ACARA, 2010). All participants were enrolled in an elementary mathematics course focusing on practical applications of mathematics such as financial arithmetic, statistics, measurement, and linear relationships. The schools encourage students who are low achieving in mathematics to take this more accessible, non-calculus-based unit, whereas students who are high achieving in mathematics are strongly encouraged to take more difficult calculus-based courses. Consequently, many students enrolling in this unit may not have previously experienced success in mathematics. All students enrolled in the course and their parents were provided with information and consent forms. Although all students completed the intervention activities as part of their normal class instruction, data were collected only for those that returned signed consent forms. Participants were randomly assigned at the classroom level to either the experimental ($n=44$) or control ($n=29$) condition, with approximately half of the classes at each school assigned to each condition. The difference in size for each condition was due to the constraints imposed by assigning conditions at the class, rather than individual, level.

### Measures

#### Spatial reasoning

**Fig. 1:**
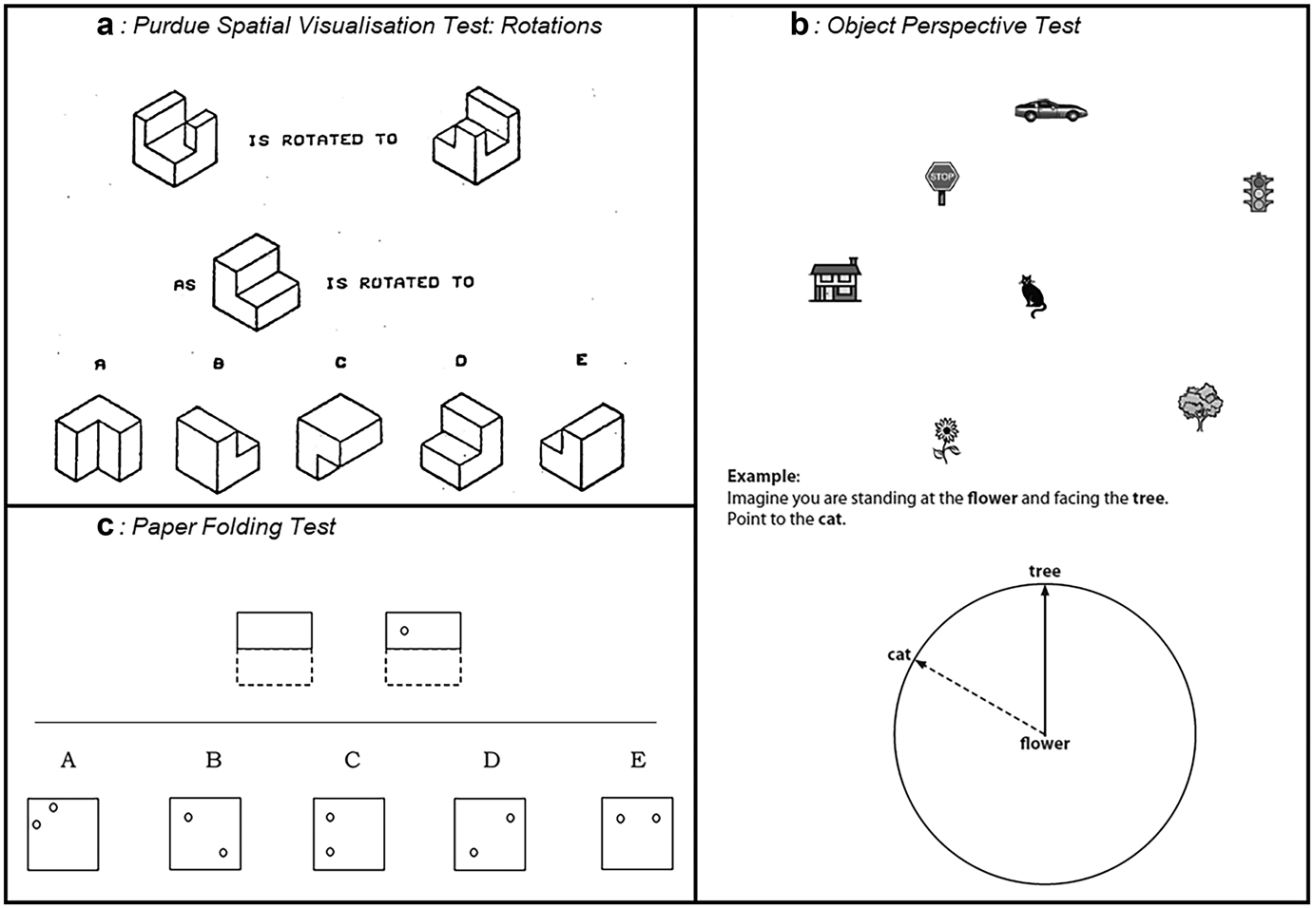
Example items from the spatial measures for: **a**, mental rotation **b**, spatial orientation **c**, spatial visualisation

In order to assess a broad range of spatial skills, the spatial reasoning measure was composed of items from three well-established spatial tests aligned with the constructs of spatial reasoning identified by Ramful et al. (2017): the Purdue Spatial Visualisation Test: Rotations (PSVT:R; Guay, 1976), the Object Perspective Test (OPT; Hegarty & Waller, 2004), and the Paper Folding Test (PFT; Ekstrom et al., 1976). A summary of sample items is provided in Fig. 1. Internal consistency of the spatial reasoning instrument used in this study was $\alpha =0.816$.

Mental rotation was measured using the PSVT:R (Guay, 1976). This test measures participants’ ability to mentally rotate an image of a three-dimensional object. Each item presents two isometric images of an object before and after undergoing one or more rotations. Participants are asked to apply the same rotations to a target item by identifying which of five response options is rotated in the same way. Throughout the test, questions become increasingly more difficult, involving larger rotations of more complex objects. Participants were given 10 minutes to complete 15 of the original PSVT:R’s 30 questions. The questions used for the shortened test were chosen to ensure maintain similar proportions with respect to the number of rotations, size of rotations, and complexity of the rotated objects in each question.

Spatial orientation was measured using the OPT (Hegarty & Waller, 2004). Participants are presented with a page displaying an array of easily recognisable objects. They are then asked to imagine themselves situated at one object, facing in the direction of a second object and to indicate the direction of a third “target” object. Participants indicate their response by marking the direction as the radius of a circle with the first object located in the middle of the circle and the second object located at 0°. In accordance with administration protocols (Hegarty & Waller, 2004), participants had 5 minutes to complete all 12 items.

Spatial visualisation was measured using the PFT (Ekstrom et al., 1976). Participants are presented with a series of images depicting a square sheet of paper being folded from 1 to 4 times, and a hole being punched through the layers of folded paper. Participants are asked to choose which of five response options would depict the locations of the holes when the paper is unfolded. Questions become increasingly complex throughout the test, with later questions involving more steps and more complex folds. In the present study, participants were given 3 minutes to complete 10 out of the PFT’s original 20 items. The questions used for the shortened version were selected to maintain similar proportions with respect to the number and complexity of the folds.

#### Mathematics

Mathematics achievement was measured using multiple-choice word problems sourced from the Australian Mathematics Competition (AMC). The AMC is an annual competition developed by the Australian Mathematics Trust and taken by more than 400,000 students each year. Twenty questions were selected to align with concepts from the Year 10 Australian Curriculum—Mathematics to ensure familiarity for Year 11 students and were piloted with a group of similar participants ($n=30$) to ensure an appropriate level of difficulty. Items where the pilot group performed below chance were removed and replaced with easier ones. Questions were chosen to align with either the Number and Algebra or Measurement and Geometry strands of the curriculum to measure the effect of the spatial reasoning intervention on different areas of mathematics. Of the 20 questions included in the final version, 11 assessed concepts from the Measurement and Geometry strand of the curriculum (e.g. nets of solids, perimeter, area, and angle) and nine assessed concepts from the Number and Algebra strand (e.g. number operations, number properties, and fractions). Internal consistency for the mathematics achievement instrument was $\alpha =0.632$. Given this measurement assesses different kinds of mathematics skills across different strands of mathematics, a lower internal consistency metric is expected (Cronbach, 1951) despite its validity (Schmitt, 1996; Taber, 2018).

### Procedure

The study utilised a pre- and post-test design. Students in the intervention group participated in the spatial reasoning intervention, while those in the control group engaged in business as usual (BAU) mathematics classes. During the program, the regular curriculum focused on the topics of rates and percentages, linear algebra, and shape and measurement. Although all students engaged with this curriculum, those in the BAU classes had slightly more class time devoted to mathematical content due to the intervention group needing to accommodate time for the spatial reasoning intervention.

Identical professional learning was provided to all teachers, regardless of condition, to control for the effect of any changes in pedagogical approaches due to teacher professional learning. Consequently, the key difference between the two groups was the spatial reasoning intervention delivered to students.

Measures of spatial reasoning and mathematics achievement were taken at the beginning and end of the program to determine the effect of the intervention. All measures were administered digitally using Qualtrics. Spatial reasoning and mathematics instruments were administered twice (as pre- and post-tests), with measures presented in the following fixed order: mental rotation, spatial orientation, spatial visualisation, and mathematics. The study was originally designed to have students complete all measures during class time. Due to COVID-19 school closures occurring at the end of the study, however, most participants (68%) completed the post-test online from home.

#### Experimental condition

Participants in the experimental condition completed modified versions of modules from Sorby et al. (2013, 2018) spatial reasoning intervention. Six of Sorby et al.'s (2013, 2018) original ten modules were chosen based on their alignment with the curriculum and Ramful et al. (2017) spatial constructs (see Table 3).

**Table 3:** Alignment between modules and Ramful et al. (2017) spatial constructs

Module	Content	Spatial alignment
Solid objects	Cut, intersection, and join operations on 3-D objects	Spatial visualisation
Isometric drawings and coded plans	Interpreting and sketching isometric drawings from different perspectives Representing isometric sketches as coded plans	Spatial orientation Spatial visualisation
Orthographic drawings	Sketching and interpreting orthographic drawings Converting between isometric and orthographic representations of 3-D objects	Spatial orientation Spatial visualisation
Single-axis rotations	Rotation of 3-D objects around a single axis	Mental rotation
Multi-axis rotations	Rotation of 3-D objects around two or more different axes in succession	Mental rotation
Flat patterns	Relating nets of solids to their 3-D representations	Spatial visualisation

Modules were delivered by classroom teachers during one timetabled mathematics lesson (approx. 60 min) per week over 6 weeks. Based on the pedagogical needs of the participants, modifications to the program focused on incorporating activities that enabled students to engage in the first three stages of the ELPSA framework. Specifically, modifications provided experiences to ground students’ understanding, opportunities for students to develop and practice using spatial language, and concrete materials where possible to help students interpret pictorial two-dimensional diagrams.

Table 4 provides an example of how the lesson on three-dimensional rotation was modified to align with the ELPSA framework, alongside the original program.

**Table 4:** Original and modified 3-dimensional rotation lessons aligned to the ELPSA framework

Element	Original program	Modified program
Experience		Revision of 2D rotations Use of blocks to build models Use of right-hand rule on real objects
Language		Definitions discussed through experience Students describe the effect of rotations using their own language
Pictorial	Definitions introduced pictorially Required to interpret 2D representations Right-hand rule explained diagrammatically	Multiple representations (photos and drawings) Gesture provided as a tool for visualisation Begin to interpret 2D representations of 3D objects
Symbolic	Introduced almost immediately	Introduced with respect to previous experiences
Application	Students solve a range of problems involving rotation around a specified axis	Students solve a range of problems involving rotation around a specified axis

The program was delivered by classroom teachers during regular mathematics classes. Teachers were provided with detailed lesson plans, scaffolding their instruction of students. Before administering the program, time was spent familiarising teachers with the lesson plans to ensure that they felt confident in delivering the content.

## Results

### Descriptive statistics

The correlations between all measured variables are presented in Table 5. Spatial reasoning pre- and post-test scores were moderately to strongly correlated with each of the pre- and post-test mathematics scores. The means and standard deviations for the intervention and control groups on each of the spatial reasoning and mathematics measures are displayed in Table 6.

**Table 5:** Correlations between measured variables

Variable	1	2	3	4	5
1. Pre-spatial					
2. Post-spatial	0.725**				
3. Pre-M and G	0.522**	0.497**			
4. Post-M and G	0.533**	0.591**	0.582**		
5. Pre-N and A	0.381**	0.387**	0.584**	0.506**	
6. Post-N and A	0.516**	0.503**	0.551**	0.602**	0.601**

*M and* G Measurement and Geometry, *N* and *A* Number and Algebra

^**^$$p<0.01$$

**Table 6:** Comparison of means between intervention and control groups

	Control					Intervention			
	Pre-test		Post-test	Pre-test				Post-test	
Measure	Mean	S.D	Mean	S.D	Mean		S.D	Mean	S.D
Spatial reasoning	0.51	0.16	0.52	0.20	0.59		0.15	0.64	0.17
Measurement and geometry	0.41	0.18	0.41	0.19	0.41		0.17	0.48	0.20
Number and algebra	0.40	0.19	0.40	0.20	0.35		0.19	0.40	0.22

### Effect of the intervention

An analysis of covariance (ANCOVA) was performed to compare the effect of the intervention on participants’ spatial reasoning post-test scores, controlling for spatial reasoning pre-test scores by including them as a covariate. The ANCOVA revealed statistically significant differences between the two groups [$F\left(\mathrm{72,1}\right)=4.051, p=0.048, d=0.48$], in favour of the intervention group.

**Fig. 2:**
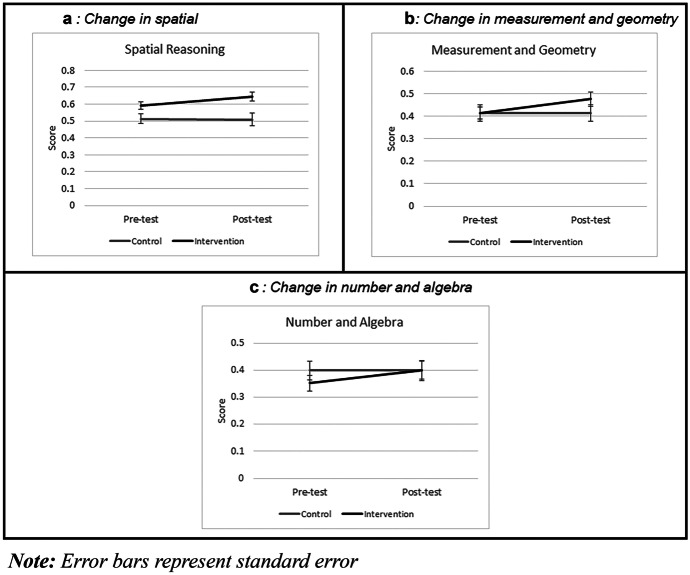
Pre- and post-test mean scores for spatial reasoning **a**, measurement and geometry **b**, and number and algebra **c**

A multiple analysis of covariances (MANCOVA) was used to analyse the effect of the intervention on the two post-test measures of mathematics achievement (measurement and geometry and number and algebra), with pre-test scores included as covariates. There was a statistically significant difference between groups on the measurement and geometry items [$F\left(\mathrm{72,1}\right)=4.154, p=0.045, d=0.45$)] in favour of the intervention group; however, there was no difference between groups for the number and algebra scores [$F\left(\mathrm{72,1}\right)=0.350, p=0.56, d=0.14$]. The changes in pre- and post-test scores on each of the three measures are displayed in Fig. 2.

## Discussion

Previous research examining the causal transfer of spatial reasoning interventions to students’ mathematics achievement have included both positive (e.g. Lowrie et al., 2017; Sorby et al., 2013) and negative (e.g. Hawes et al., 2015) results. The present study accounts for these mixed findings by considering the multidimensional nature of spatial reasoning and mathematics and the role of a strong pedagogical framework in supporting transfer. Our main finding was that spatial training was effective in improving spatial skills for lower-achieving Year 11 students, a previously unexplored population and that these gains transferred to improved performance on a broad range of measurement and geometry questions.

These findings demonstrate that transfer between spatial reasoning training and mathematics achievement can be achieved. Here, we embedded a spatial reasoning intervention within a mathematics classroom that incorporated different kinds of spatial reasoning skills, extended over a longer timeframe, and used a strong pedagogical framework. Although more research is required to determine if these attributes have a differential influence on transfer, in sum, this approach explains previous varied findings. Interventions that have taken place over short timeframes (e.g. Gilligan et al. 2020) or focused on only a single spatial skill (e.g. Hawes et al., 2015) have shown narrow or no transfer. In contrast, studies demonstrating broad transfer, such as the present study, have taken place over a longer timeframe and incorporated a wider variety of spatial skills (e.g. Hawes et al., 2017; Lowrie et al., 2017; Sorby et al., 2018). Below, we consider our findings in the context of these key attributes.

In considering the multidimensional nature of mathematics and spatial reasoning, the present study found transfer on mathematics tasks that were more closely aligned to the intervention (i.e., spatial reasoning supported geometry and measurement). By way of example, geometric reasoning (e.g. identifying rotation, reflection, and symmetry of objects) implicitly requires mental manipulation of spatial information (Clements & Battista, 1992; Jones, 2001). Subsequently, improving spatial reasoning in the current study may have provided students with the prerequisite skills required to engage in more complex geometric reasoning (Mamolo et al., 2015). Previous research has consistently shown that spatial skills are key predictors in geometry (e.g. Battista et al., 1982; Delgado & Prieto, 2004) and that spatial learning transfers to performance on geometry tasks (Hawes et al., 2017; Lowrie et al., 2017, 2018a, 2019).

In contrast, we did not find that spatial learning transferred to number and algebra items. This is aligned with previous research, which has found that mental rotation (a spatial reasoning skill) predicted performance on geometry and not algebra (Kyttälä & Lehto, 2008; Weckbacher & Okamoto, 2014). Interestingly, Kyttälä and Lehto (2008) also found that visuospatial working memory (how much spatial information can be kept active in the mind) was predictive of algebra and not geometry. Newcombe et al. (2019) offer an explanation by pointing to differences inherent in the task demands. It is the case that geometry involves reasoning about transformations (e.g. imagining objects rotate and visualising change), whereas algebra can involve mental operations (Heathcote, 1994), which requires keeping in mind the location of numbers and variables, relevant operations, and completing mental arithmetic.

Previous research on transfer between spatial training and number and algebra concepts have had mixed results, with some finding transfer (e.g. Hawes et al., 2017; Lowrie et al., 2021) and others not (e.g. Hawes et al., 2015; Lowrie et al., 2017). Duffy (2020) also highlights the role of task demands in explaining transfer, finding that spatial training transferred only to algebraic word problems that required the interpretation of key spatial information and not to algebraic word problems that could be solved procedurally. This may explain the mixed results—for example in the Lowrie et al. (2017) study, students were required to solve non-geometric word problems, whereas in Hawes et al.'s (2017) study, the number problems were procedural. Although the number and algebra items in the current study could be solved using a spatial strategy, on reflection, not all items were dependent on the interpretation of spatial information. Notably, visuospatial working memory also supports spatial reasoning skills (Heathcote, 1994), and so spatial skills training may indirectly improve algebra if the training also improved visuospatial working memory.

Another key component of the current study was its use of a pedagogical framework: the ELPSA model (Lowrie et al., 2018b). Most studies examining transfer between spatial training and mathematics achievement do not explicitly consider pedagogy (e.g. Bower et al., 2020; Cheng & Mix, 2014; Cornu et al., 2019; Hawes et al., 2015; Mix et al., 2020; Rodán et al., 2019; Sorby et al., 2013, 2018; Xu & LeFevre, 2016), and, with the exception of Sorby et al. (2013, 2018), these studies simply use some form of corrective feedback as the basis of training. Notably, most of these studies do not achieve transfer from spatial training to mathematics (Cornu et al., 2019; Hawes et al., 2015; Rodán et al., 2019; Xu & LeFevre, 2016) or find only narrow transfer (Cheng & Mix, 2014). The three studies that did find broader transfer (Bower et al., 2020; Mix et al., 2020; Sorby et al., 2013, 2018) engaged children in a range of spatial tasks extended over longer periods of time (ranging from 3 weeks to a whole semester). Where pedagogy has been considered (Hawes et al., 2017; Lowrie et al., 2017, 2019), such as the current study, all find transfer. Together, these findings likely reflect a gap between cognitive science and educational research (Hawes et al., 2017) and highlight the role of pedagogy in scaffolding transfer.

The current study utilised the ELPSA pedagogical framework in the design of the intervention. Investigation of the individual contributions of spatial training and pedagogical approach to mathematics achievement was beyond the scope of the study. Future research should assess the differential contribution of spatial training and pedagogical approaches on mathematics achievement.

Although the current study, and Lowrie et al. (2017, 2019), found success using the ELPSA model, it is possible that other pedagogical frameworks would be equally effective at achieving transfer. For example, Hawes et al. (2017) used a modified version of the Japanese Lesson Study (Lewis et al. 2006), which involved their research team collaborating extensively with educators and students to iteratively design spatial lessons. Pedagogical considerations of Hawes et al. (2017) and ELPSA in the current study both involved several of the same attributes: building educator capacity around spatial reasoning, providing learning tools that can be flexibly administered, use of constructivist approaches (e.g. inquiry-based activities using hands-on materials), and building increasingly complex spatial skills over an extended period of time. Future research should examine if a particular pedagogical framework is better suited for spatial learning. We hypothesize that the ELPSA model would be a strong candidate because it explicitly includes spatialized steps (i.e., using spatial language and creating pictorial representations).

## Limitations

The present study aimed to achieve a high level of ecological validity, by having the program delivered by educators within the classroom. A small number of schools were chosen so that the researcher was able to closely support the educators as they implemented the program. As a result, the relatively small sample size limits our ability to examine nested effects (e.g. school-level factors) and the generalisability of the program across populations. It is also possible that the spatial intervention had effect on students’ number and algebra performance, but we lacked sufficient power to detect differences between the intervention and control groups. Though notably, our findings are consistent with previous research also showing no connection between spatial reasoning training and number and algebra topics (e.g., Kyttälä & Lehto, 2008; Newcombe et al., 2019; Weckbacher & Okamoto, 2014). More research should be done with larger sample sizes to explore the effect of spatial training on different mathematical areas for this age group. The participating schools from the present study primarily served families from middle to higher SES (ACARA, 2020). However, we hypothesise that the ELPSA-enriched Sorby program would also support mathematics learning for children from lower SES given that children from lower SES backgrounds make larger gains in transferring improved spatial skill to mathematics outcomes (Bower et al., 2020). Future research should examine the efficacy of the program across a wider range of populations at scale. In addition, future research should examine how affective factors (e.g. mathematics or spatial anxiety) interact with the effectiveness of spatial interventions supporting transfer to mathematics achievement.

## Implications and conclusions

The current study demonstrates that spatial learning can causally support mathematics achievement. In developing spatial learning programs, educators and researchers should consider the relations between specific spatial skills and mathematics tasks as well as the incorporation of a pedagogical framework to scaffold transfer. We have identified three roles of pedagogical frameworks within spatial intervention studies. First, pedagogical frameworks can be used as an analytic tool to examine the likely utility of an intervention to achieve transfer (as was done in the current study when modifying the program for use with Year 11 students). Pedagogical frameworks can also scaffold transfer by supporting educator delivery of spatial learning and engaging children in ways they naturally learn.

Pedagogical frameworks, such as ELPSA, in spatial learning may also support educators with both low and high spatial reasoning skills in delivering spatial training to students. Most secondary STEM educators have higher than average spatial reasoning skills (Atit et al., 2018), which may result in them not being aware of barriers low-spatial students may face. The mismatch in spatial skill between low-achieving students and their teachers represents a challenge to the high-school context. ELPSA requires educators to begin by building shared experiences in basic concepts, moving stepwise from more concrete examples to more abstract applications. In contrast, educators with low-spatial reasoning skills frequently avoid using spatial tools (Atit & Rocha, 2021; Otumfuor & Carr, 2017) and may avoid teaching spatial topics altogether (Gunderson et al., 2013). The ELPSA framework has been shown to provide educators with increased confidence and interest in teaching spatial learning (Resnick & Logan, 2021).

Previous research has focused on high-achieving mathematics students (e.g. Miller & Halpern, 2013; Sorby et al., 2013, 2018) or random samples (e.g. Cheng & Mix, 2014; Gilligan et al., 2020; Lowrie et al., 2019); however, relatively low-achieving students may have additional pedagogical needs beyond those of high-achieving students. For example, Reinhold et al. (2020) found that low-achieving students benefited much more from a broad, conceptually focused fractions curriculum when provided with adaptive scaffolding to support their learning. By contrast, high-achieving students do not generally require the same level of scaffolding to benefit from the same curriculum (Lowrie, 2020). The current study shows the utility of the ELPSA framework for scaffolding transfer for students who have less experience with advanced mathematics and are not pursuing future careers in STEM. Reaching this population is crucial, as students with weaker mathematics skills stand to gain the most from interventions such as these.

## Declarations

### Research involving human and animal participants

The study involved research with human participants (senior secondary high-school students. Ethics was provided by the University of Canberra’s IRBHREC (ID: 20202105).
